# Variation in acute care burden and supply across diverse urban settings

**DOI:** 10.1186/cc12427

**Published:** 2013-03-19

**Authors:** S Murthy, S Austin, H Wunsch, NK Adhikari, V Karir, K Rowan, ST Jacob, J Salluh, F Bozza, B Du, Y An, B Lee, F Wu, C Oppong, R Venkataraman, V Velayutham, D Angus

**Affiliations:** 1 Hospital for Sick Children, Toronto, Canada; 2 University of Pittsburgh, PA, USA; 3 Columbia University, New York, NY, USA; 4 University of Toronto, Canada; 5 Intensive Care National Audit & Research Centre, London, UK; 6 University of Washington, Seattle, WA, USA; 7 D'OR Institute for Research and Education, Rio de Janeiro, Brazil; 8 Oswaldo Cruz Foundation, Rio de Janeiro, Brazil; 9 Peking Union Medical College Hospital, Beijing, China; 10 Peking University People's Hospital, Beijing, China; 11 Komfo Anokye Teaching Hospital, Kumasi, Ghana; 12 Apollo Hospital, Chennai, India; 13 Stanley Medical College Hospital, Chennai, India

## Introduction

The World Bank has warned that the rapid growth of the world's urban population can only be accommodated safely if cities adequately develop key infrastructure, such as the provision of acute care resources. Yet, even basic descriptive information on urban acute care supply and demand is extremely limited. We therefore conducted a pilot assessment across seven diverse urban settings across the world.

## Methods

We selected a convenience sample of seven large cities with varying geographical and socioeconomic characteristics: Boston, Paris, Bogota, Recife, Liaocheng, Chennai, and Kumasi. To estimate acute care supply, we developed an instrument to collect data on acute and critical care infrastructure. We collected data from municipal authorities and local research collaborators. We expressed the burden of acute disease as the number of deaths due to acute illnesses, estimated from the 2008 Global Burden of Disease Study. Results were expressed as acute care supply and acute deaths per 100,000 population and acute care supply per 100 acute deaths.

## Results

The supply of hospital beds varied from 72.4/100,000 population in Kumasi to 245.8/100,000 in Boston. ICU beds with capacity for invasive mechanical ventilation and intensive nursing services ranged from 0.4/100,000 in Kumasi to 19/100,000 population in Boston. The number of ambulances varied 70-fold between cities. The gap between cities widened when demand was estimated based on disease burden, with a 70-fold difference between cities in ICU beds/acute deaths. In general, most of the data were unavailable from municipal authorities.

## Conclusion

The provision of acute care services, a key aspect of urban infrastructure, varied substantially across the seven diverse urban settings we studied. Furthermore, the local municipal authorities generally appeared to have little knowledge of their acute care infrastructure, with implications for future planning and development.

